# FAIR chemical structures in the Journal of Cheminformatics

**DOI:** 10.1186/s13321-021-00520-4

**Published:** 2021-07-07

**Authors:** Emma L. Schymanski, Evan E. Bolton

**Affiliations:** 1 Luxembourg Centre for Systems Biomedicine (LCSB), University of Luxembourg, 6 avenue du Swing, 4367 Belvaux, Luxembourg; 2 National Center for Biotechnology Information, National Library of Medicine, National Institutes of Health, Bethesda, MD 20894 USA

**Keywords:** Open science, Open access, Chemical information, Cheminformatics, Chemical database, Chemical deposition, FAIR, Supplementary material, Data archive, Open repository

## Abstract

The ability to access chemical information openly is an essential part of many scientific disciplines. The Journal of Cheminformatics is leading the way for rigorous, open cheminformatics in many ways, but there remains room for improvement in primary areas. This letter discusses how both authors and the journal alike can help increase the ***FAIR***ness (Findability, Accessibility, Interoperability, Reusability) of the chemical structural information in the journal. A proposed chemical structure template can serve as an ***interoperable*** Additional File format (already ***accessible***), made more ***findable*** by linking the DOI of this data file to the article DOI metadata, supporting further ***reuse***.

**Supplementary Information:**

The online version contains supplementary material available at 10.1186/s13321-021-00520-4.

## Main Text

The Journal of Cheminformatics (hereafter JCheminform) contains chemical structures in nearly every published article. However, if readers want to find which articles contain a particular structure, or download the structures from a particular article, it is not possible unless the author makes them readily available. Even then, each author might do this differently, increasing the downstream effort by data users.

JCheminform is helping lead the way for open and ***FAIR*** (Findable, Accessible, Interoperable, Reusable [1]) chemical informatics. For example, all Additional Files in JCheminform articles are uploaded into FigShare [2], thus helping make them more ***accessible***. However, JCheminform can further its ***FAIR***ness leadership by enabling two key initiatives for its chemical structure content: establishing a consistent approach to reporting chemical structures per article by introducing a chemical structure data template for Additional Files and enhancing the article DOI metadata to link back to this chemical structure data file DOI in FigShare.

While bioinformatics, crystallography and other scientific fields require data to be published in an open repository, there is no such requirement for chemical information; yet, there is movement in this direction. Some publishers (e.g., Springer Nature [3]) have established automated submission of chemical structure content to open archives (such as PubChem [4]) using name-entity approaches. Some primary chemistry journals have established standard templates for reported chemical structure and associated content (e.g., Journal of Medicinal Chemistry [5]) or by means of direct chemical structure submission into an open archive (e.g., Nature Chemical Biology [6]).

If chemical structures found in articles are provided in a machine-readable way, e.g., via a template file, then the chemical structures are more ***interoperable*** and can be readily ***reused*** by researchers and directly integrated by chemical-centric resources. If the journal supports ***findability*** of this chemical data, for example by indicating its availability in the article DOI metadata or by providing a mapping file (containing the article DOI and the FigShare DOI of the chemical data file) on the journal website, then scientists and chemical resources can readily locate the chemical structure content. By improving the ***findability*** and ***interoperability***, the barriers for ***reuse*** are lowered (see e.g., [7]). These author contributions, especially with valuable additional annotation information, are essential to fill gaps in the current chemical knowledge [8]. Thus, we believe it is time for JCheminform to take these logical next steps towards Open Science and help authors provide Open and ***FAIR***er chemical data.

## Chemical structure data template file

Authors should be encouraged to submit their chemical structure information using a standard template as “Additional Files” with their manuscript in either CSV (*.csv), TSV (*.tsv) or SDF (*.sdf) format.

For CSV/TSV, the header (first row) indicates the data content of each column; each subsequent row corresponds to a complete chemical record description: chemical structure, chemical names, identifiers, comments, and any other data the authors wish to provide (as additional columns). The case-insensitive template CSV/TSV column headers (or SDF SD fields) are: ***SMILES***, ***InChI***, and ***InChIKey*** for chemical structure; ***Name*** and ***Synonym*** for chemical names; and ***Comment*** for textual comments. Any additional columns headers, *e.g.*, for data, further identifiers or metadata, are up to the author. The ***Synonym*** and ***Comment*** columns may be provided more than once per record.

**Fig. 1:**
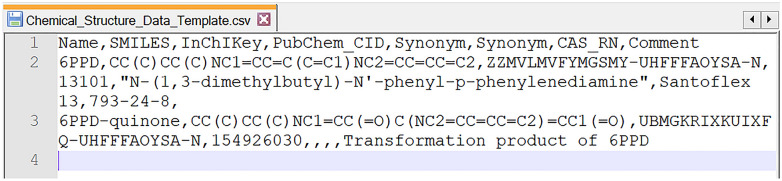
Example chemical structure data template file for **interoperable** chemical information [9]

The author-submitted template file should contain at least one of the following columns: ***SMILES***, ***InChI***, ***Name*** or ***InChIKey***. The ***Name*** column corresponds to a single primary name for the chemical structure. Each ***Synonym*** column corresponds to an additional chemical name (one name entry per column). Each ***Comment*** column can be added to provide additional text that may be important to the downstream user. Authors can also provide additional CSV/TSV columns or SDF SD fields of information to describe their chemical substances (with unique, descriptive headers) for additional context. Chemical database identifiers or registry numbers could be included in this manner, or as a ***Synonym***. See Fig. 1 and Additional file 1 [9] as an example.

Note that chemical records indicating chemical structure with only ***InChIKey*** or ***Name*** will not contain sufficient information to describe a chemical structure, and *can only be mapped to existing entries* in destination resources. Batch services are available (e.g. from PubChem [4, 10] or CompTox [11, 12]) for authors to add e.g.* SMILES* and/or ***InChI*** to their records.

## Closing

The era of Open and ***FAIR*** chemical science is upon us. Providing standard templates and guidance for authors to submit their chemical data in an ***interoperable*** manner that is tagged in a ***findable*** way by the journal will help close the gaps in databases, raise the visibility of their scientific contributions, improve the machine readability, help authors meet funding data sharing requirements and increase the utility of information in JCheminform. We strongly believe that authors and readers alike will greatly appreciate this ***FAIR***ifying value-add towards Open Science.

## Supplementary Information

**Additional file 1.** Example chemical structure data template file for ***interoperable*** chemical information.

## Data Availability

The chemical structure data submission template is provided as Additional file 1 and here [9].

## Abbreviations

CAS_RN: Chemical Abstract Service Registry Number
CSV: Comma-Separated Values
DOI: Digital Object Identifier
FAIR: Findable, Accessible, Interoperable, Reusable
InChI: The IUPAC International Chemical Identifier
InChIKey: The hashed form of the InChI
JCheminform: Journal of Cheminformatics
NORMAN-SLE: NORMAN Network Suspect List Exchange
PubChem_CID: PubChem Compound Identifier
SDF: Structure-Data File
SMILES: Simplified Molecular Input Line Entry System
TSV: Tab-Separated Values
